# Dog and Cat Contact as Risk Factor for Human Toxocariasis: Systematic Review and Meta-Analysis

**DOI:** 10.3389/fpubh.2022.854468

**Published:** 2022-06-28

**Authors:** Yslla Fernanda Fitz Balo Merigueti, Rogerio Giuffrida, Rodrigo Costa da Silva, Louise Bach Kmetiuk, Andrea Pires Dos Santos, Alexander Welker Biondo, Vamilton Alvares Santarém

**Affiliations:** ^1^Graduate College of Physiopathology and Animal Health, University of Western São Paulo (UNOESTE), Presidente Prudente, Brazil; ^2^Graduate College of Animal Science, University of Western São Paulo (UNOESTE), Presidente Prudente, Brazil; ^3^Laboratory of Cell Biology, Instituto Carlos Chagas, Fundação Oswaldo Cruz, Curitiba, Brazil; ^4^Department of Comparative Pathobiology, College of Veterinary Medicine, Purdue University, West Lafayette, IN, United States; ^5^Department of Veterinary Medicine, Federal University of Paraná (UFPR), Curitiba, Brazil

**Keywords:** companion animals, epidemiology, larva migrans, zoonoses, *Toxocara* spp.

## Abstract

Toxocariasis, a neglected parasitic zoonosis with worldwide distribution, has been reportedly associated to different risk factors in several epidemiological and meta-analysis studies. However, dog and cat contact (environmental and animal exposure) as isolated associated risk factor for children and adults remains to be fully established. Accordingly, the present meta-analysis has aimed to directly assess dog and cat contact for toxocariasis seropositivity in under-18 and adult persons, using a survey strategy of PubMed/Medline, Embase, Scopus and Scielo Databases, from January 2009 to December 2021. A meta-analysis model of random effects was applied to estimate *odds ratio* (OR) with 95% Confidence Interval (CI). The statistical heterogeneity was evaluated by the Cochran Q-Test and *I*^2^ values. A total of 41 transversal studies (*n* = 20.515 individuals) from different geographic regions (classified by the World Health Organization) were included herein. In overall, 1,882/13,496 (13.95%; 95% IC = 13.4–14.5) youngers and 513/7.019 (7.3%; 95% CI = 6.7–7.9) adults in contact with dogs or cats were serologically reagent for anti-*Toxocara* antibodies. Association of dog and cat contact was observed only in youngers, with both dogs (OR = 1.53; *p* < 0.0001) and cats (OR = 1.64; *p* = 0.0001). In addition, association of dog and contact and serology was statistically significant in populations of Americas (OR = 1.37; 95% CI = 1.1–1.7), Middle East (OR = 2.9; 95% CI = 1.6–5.1) and West Pacific (OR = 1.6; 95% IC = 1.3–1.9). In conclusion, contact with dogs and cats, particularly by younger individuals and in regions such as Americas, Middle East, and West Pacific, should be always a public health concern for toxocariasis. Moreover, dogs and cats should be periodically dewormed, washed and hair cleaned prior to contact with youngers. Finally, robust statistical results herein may serve as basis for future strategies and preventive measures for safer dog and cat contact.

## Introduction

Toxocariasis is a worldwide parasitic zoonosis caused by nematodes *Toxocara canis* and *Toxocara cati* from dogs and cats as definitive hosts, respectively (1, 2). Classified amongst the top six parasitic infections of priority to public health by the World Health Organization and Centers of Disease Control (3), toxocariasis global seropositivity has been estimated in 19% by a recent meta-analysis study (CI 95% = 16, 6–21,4%) (4).

Primarily considered as geo-zoonosis, the main toxocariasis transmission occurs by accidental ingestion of larvae in eggs from the soil (5). In addition, fecal-oral transmission may occur after intake of contaminated soil with embryonated eggs of *Toxocara* spp., particularly in gardens, sandboxes, public squares, and parks (6, 7). The global prevalence of environmental contamination by eggs of *Toxocara* spp. has been estimated of 21% (CI = 16–27%) by another recent meta-analysis study (8).

Pet access to outdoors areas of human gathering and closer human: pet contact may predispose higher soil contamination and lead to higher human exposure to *Toxocara* spp. (9, 10). Although uncommon, transmission may also occur by ingestion of *Toxocara* spp. eggs from contact with non-dewormed dogs and cats (11–13).

Systematic reviews and meta-analysis studies have recently established clinical onset of toxocariasis, including disease association to respiratory (14, 15), neurological (16, 17), and dermatological disorders (18). Furthermore, prevalence has been established worldwide, including different regions such as Europe (19) and Western Pacific (20).

The concept of One Health highlights the interconnection among human, animal and environmental health, and the importance of multidisciplinary collaborations to address challenges in global health (21). Despite evidence of human recurrent exposure to *Toxocara* spp., comprehensive studies should be conducted to fully establish the impact on public health. In such scenario, One Health approach has been reportedly indicated as a vital tool to confront the complexity of human toxocariasis worldwide (22, 23).

Although contact with companion animals (dogs and cats) has already been established as risk factor for toxocariasis (4), age groups have shown different exposure characteristics, mostly related to immunological system, hygiene and food habits (24). Even so, no study to date has focused on risk association of disease to youth and adulthood as independent approaches. Accordingly, the present study aimed to individually assess younger (under-18) and adult age groups and contact with dogs and cats as associated risk factors for toxocariasis.

## Methods

### Search Strategy and Selection Criteria

The study herein has used components of the Preferred Reporting Items for Systematic Reviews and Meta-Analyses (PRISMA) (Supplementary Material), besides applying the guidelines for conception, execution, and interpretation of results (8, 25). The search strategy was based on screening of scientific articles that evaluated seroprevalence of toxocariasis in persons with dog or cat contact, from January 2009 to June 2019.

The search was performed in different databases including PubMed / Medline, Embase, Scopus e Scielo. A combination of terms was used, resulted in the following Mesh of (toxoc^*^) AND (prevalence OR seroepidem^*^ OR serol^*^ OR seroprevalence) AND (risk factor^*^), and was presented (Table 1). Literature surveyed in the present study included English, Spanish and Portuguese. Mostly to avoid overlapping information, theses and dissertations data were not assessed.

**Table 1:** Search strategy in different databases utilized for meta-analysis for assessment of contact with dogs and cats as associated risk factor for toxocariasis from 2009 to 2021.

**Database**	**Search strategy**
Embase	((toxoc*) AND (prevalence OR seroepidem* OR serol* OR seroprevalence) AND (risk factor*))
PubMed	((toxoc*) AND (prevalence OR seroepidem* OR serol* OR seroprevalence) AND (risk factor*)) AND (“2009/01/01”: “2021/12/31”))
Scielo	(toxoc*) AND (prevalence OR seroepidem* OR serol* OR seroprevalence) AND (risk factor*) AND year_cluster (“2021” OR “2020” OR “2019” OR “2018” OR “2017” OR “2016” OR “2015” OR “2014” OR “2013” OR “2012” OR “2011” OR “2010” OR “2009”)
Scopus	((toxoc*) AND (prevalence OR seroepidem* OR serol* OR seroprevalence) AND (risk AND factor*)) AND (limit to pubyear 2021 to 2009))

Following removal of duplicates and screening of titles and abstracts, assessment of full texts was thoroughly performed for inclusion or exclusion in the meta-analysis study, with a final screening for solving conflicts, as previously described (8). Inclusion criteria included studies assessing contact, with adults or under-18 individuals, involving dogs and cats and that used serological methods for detection of anti-*Toxocara* (IgG) antibodies; geographical and idiom restrictions were not applied.

Articles failing to make the inclusion criteria were discharged, such as studies with comorbidities and/or without control groups, evaluating only dogs and cats, with no identification of animal species as risk factor, with no age stratification, case report or series of reports, clinical cases of diagnosed infection by *Toxocara* spp., and reviews, systematic reviews, or meta-analyses.

### Data Extraction

Data extraction was independently performed following examination of eligibility criteria, with information gathered in a commercially available software (Excel, version 2016, Microsoft Co., Redmond, WA, USA). Data registered for analysis included author name, year of publication, country where the study was performed, age of population (children, under-18, adults, above-18), sample size, pet species assessed as associated risk factor (dog and/or cat), number of seropositive/seronegative individuals with and without contact with companion animals.

### Meta-Analysis

Estimative of toxocariasis seroprevalence in individuals with and without contact with companion animals were calculated using a model of random effects with Confidence Interval (CI) of 95%, providing an overall estimative. In addition, age population and region where the study was performed, along with correspondent geographical coordinates, were also calculated.

Heterogeneity among studies was calculated by the Q-test of Cochran, which has considered as significant the values of *p* < 0.05. Values of I^2^ ≥50% found were used to define the significant level of heterogeneity (8). Analysis of meta-regression were performed according to several parameters including country, animal species involved, human population age, presence of absence of contact to dog and/or cat, and seropositivity/seronegativity to anti-*Toxocara* (IgG) antibodies.

Contact with dogs and/or cats was considered in environmental and animal exposure, when individuals owned dogs or cats, had contact to dogs or cats, kept dogs or cats, played with dogs or cats, had intra-domiciliary or peri-domiciliary presence of dogs or cats, had dogs or cats at home, fed dogs or cats, raised dogs or cats, had contact with dogs or cats.

Assessment of potential associated risk factor related to seroprevalence of anti-*Toxocara* (IgG) antibodies and contact with dogs and cats was made by evaluation of *odds ratio* (OR) and Confidence Interval (CI) of 95%. Forest plots were constructed to present results of meta-analysis in a schematic fashion and with funnel graphics (each study was represented by a proportional effect size), and it was performed to assess publication bias (26, 27).

In order to evaluate the studies which overly contribute to the heterogeneity in meta-analysis, sensitivity analysis were conducted by Baujat plots (28).

Statistical analyses were conducted utilizing a commercially available package (29), implemented in the R Project (30). Results of statistical analyses were considered significant when *p* < 0.05.

## Results

### Study Characteristics

**Figure 1:**
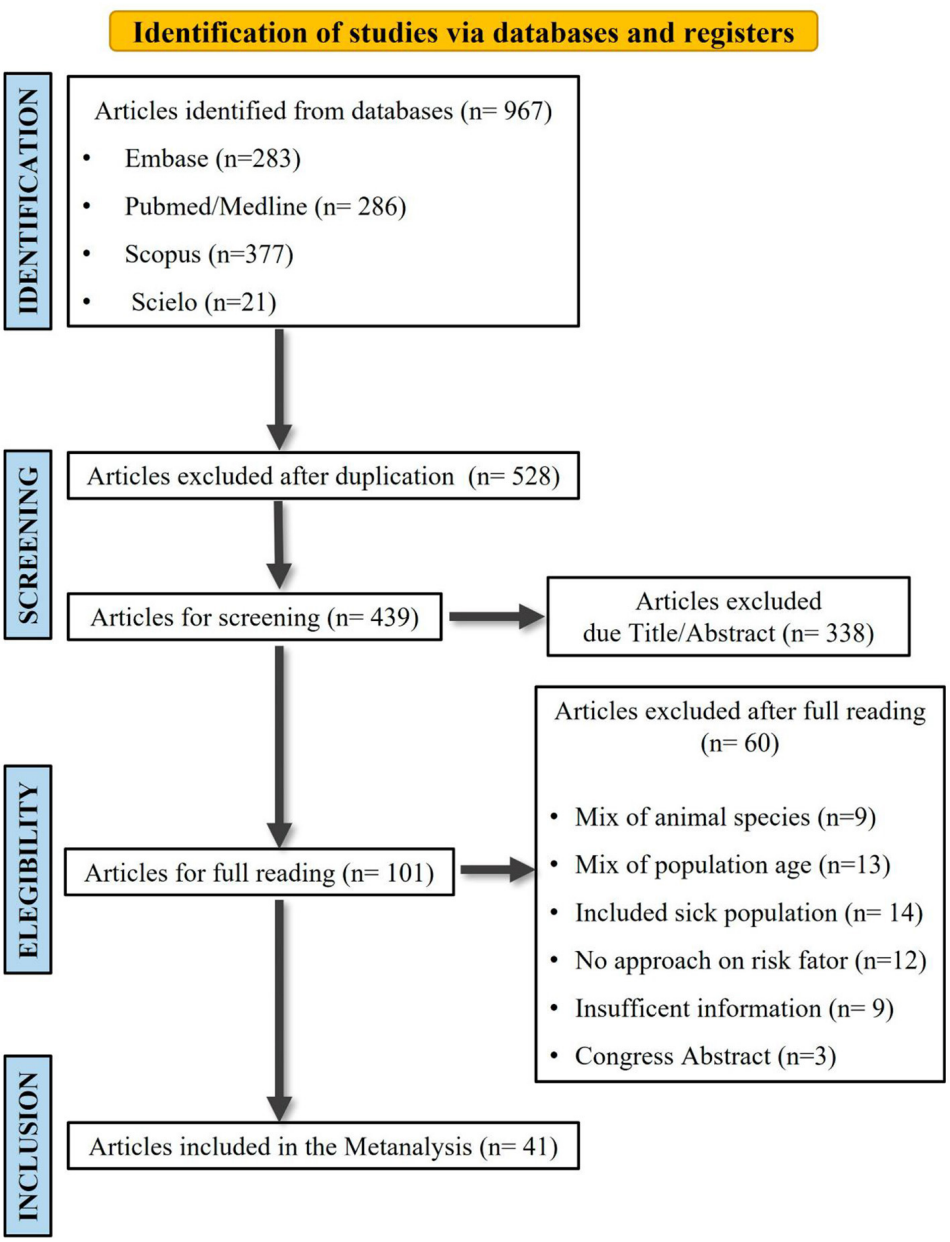
Flow-chart representing study search and selection strategy on seropositivity of (IgG) anti- *Toxocara* antibodies and contact with dogs and/or cats in articles of different databases included in the meta-analysis study, from 2009 to 2021.

Initial bibliographical search comprised a total of 967 scientific articles, followed by application of eligibility criteria, and resulting in a final inclusion of 41 articles for the meta-analysis study (Figure 1).

Selected articles were from studies conducted in 13 different countries (Austria, Brazil, Colombia, China, Equator, Iran, Mexico, Norway, Nigeria, Serbia, Taiwan, Thailand, and Venezuela), representing different regions of the World Health Organization (OMS, 2021). Countries with most reports included Brazil with ten, Iran with six, Mexico with four, and Venezuela with three; followed by China and Taiwan with two each and the remaining countries with one study each. Regarding to geographic regions, 21/41 (51.2%) were taken in the Americas, 9/41 (22.0%) in Middle East, 5/41 (12.2%) in the Western Pacific, 3/41 (7.3%) in Europe, 2/41 (4.9%) in Africa, and one Southeast Asia 1/41 (2, 4%).

The 41 selected articles represented a total population of 20,515 individuals, with an overall prevalence of 24.1% (4,948/20,515; CI 95% = 23.5–24.7%) for anti-*Toxocara* antibodies. Considering the total population among studies a total of 6,826 (33.3%) participants were from the Americas, 6,113 (29.8%) from Western Pacific 4,859 (23.7%) from Middle East, 1,819 (8.9%) from Europe, 721 (3.5%) from Africa, and 177 (0.9%) from Southeast Asia.

Regarding to the presence of anti-*Toxocara* antibodies the highest prevalence was observed in the Africa with 497/721 (64.5%; CI 95% = 65.4–72.3%), followed by Americas with 2,774/6,826 (40.6%; CI 95% = 39.0–41.2%), Western Pacific with 865/6,113 (14.5%; CI 95% = 13.3–15.1%), Middle East with 548/4,859 (11.3%; CI 95% = 10.4–12.2%) and Europe with 161/1,819 (8.9%; CI 95% = 7.6–10.3%). Only one study was carried out in Southeast Asia resulting in a prevalence of 103/177 (58.2%/ CI 95% = 50.6–65.6%). The highest prevalence was observed in a serosurvey in Africa (92.5%) and the lowest in Iran (1.4%). Regarding geographic location, the highest prevalence was observed between latitudes of 0 and 20° (41.1%).

Assessment of having direct contact with dogs or cats showed the highest frequency in the population of Americas with 1,717/6,826 (25.2%; CI 95% = 24.1–26.2%), followed by Africa with 59/721 (8.2%; CI 95% = 6.3–10.4%), Western Pacific with 360/6,113 (5.89%; CI 95% = 5.3–6.5%), Middle East with 179/4,859 (3.7%; CI 95% = 3.2–4.3%), and Europe with 43/1,819 (2, 4%; CI 95% = 1.8–3.2%). In the solely Asian included study, 37 out of 177 (20.9%) individuals reported having contact with dog/cat.

A total of 27/41 (65.9%) studies were conducted with children and under-18 participants, while 12 (27/41; 29.3%) studies were conducted with adults, and two (4.9%) with adults and children independently. Overall prevalence of 3,665/13,496 (27.2%; CI 95% = 26.4–27.9%) was observed in under-18 participants, while 1,283/7,019 (18.3%; CI 95% = 17.4–19.2%) in adults.

Most of the studies (24/41; 58.5%) used indirect ELISA for detection of anti-*Toxocara* antibodies followed by 4/41 (9.8%) studies with Western blot and 5/41 (12.2%) using both diagnostic tests; commercial ELISA kits were used in the remaining 8/41 (19.5%) articles.

### Meta-Analysis Results

**Figure 2:**
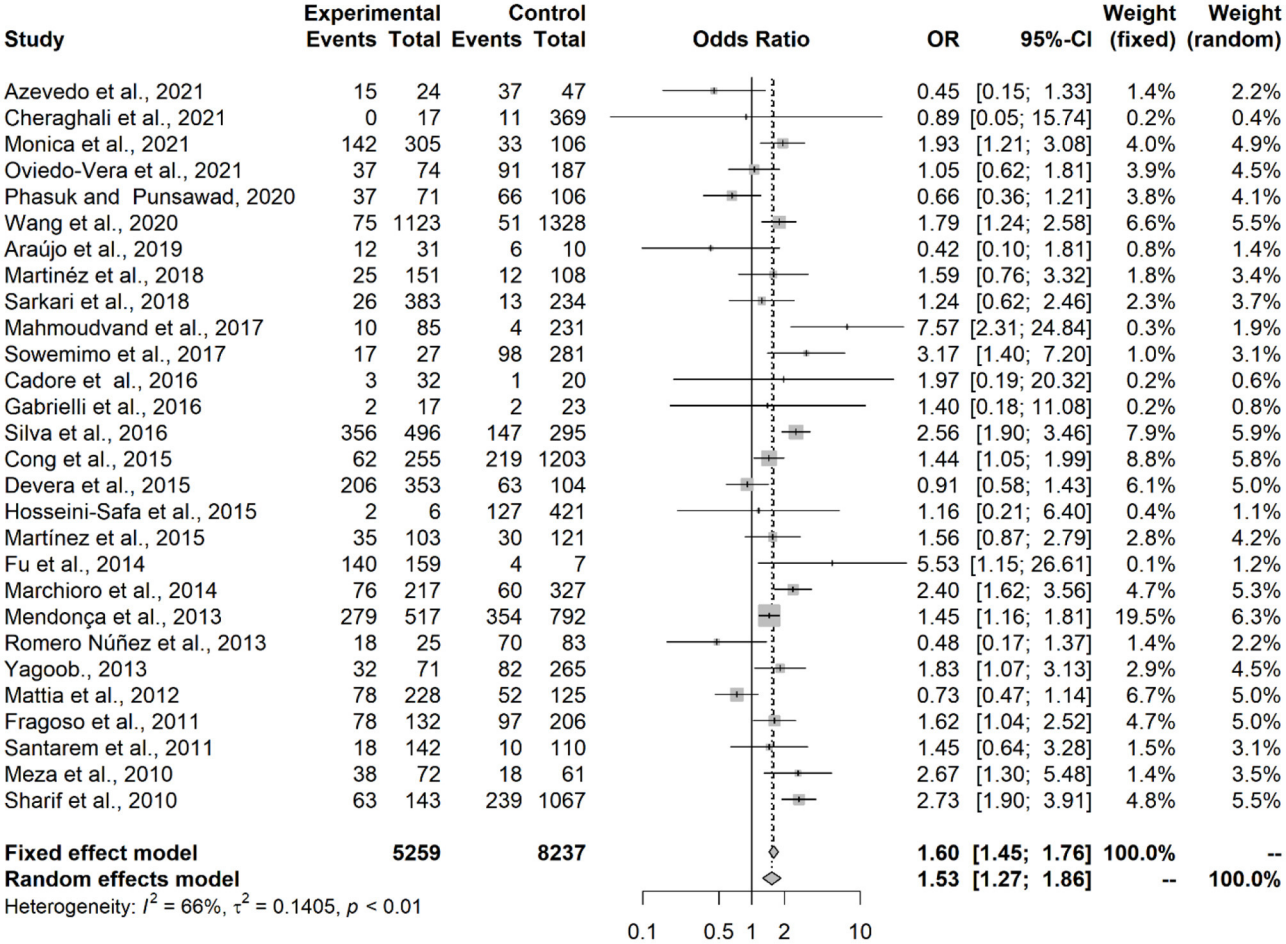
Forest plot for assessment of *odds-ratio* of influence of contact with dogs in the frequency of anti-*Toxocara* antibodies in under-18 participants, according to selected articles used in the meta-analysis from 2009 to 2021.

**Figure 3:**
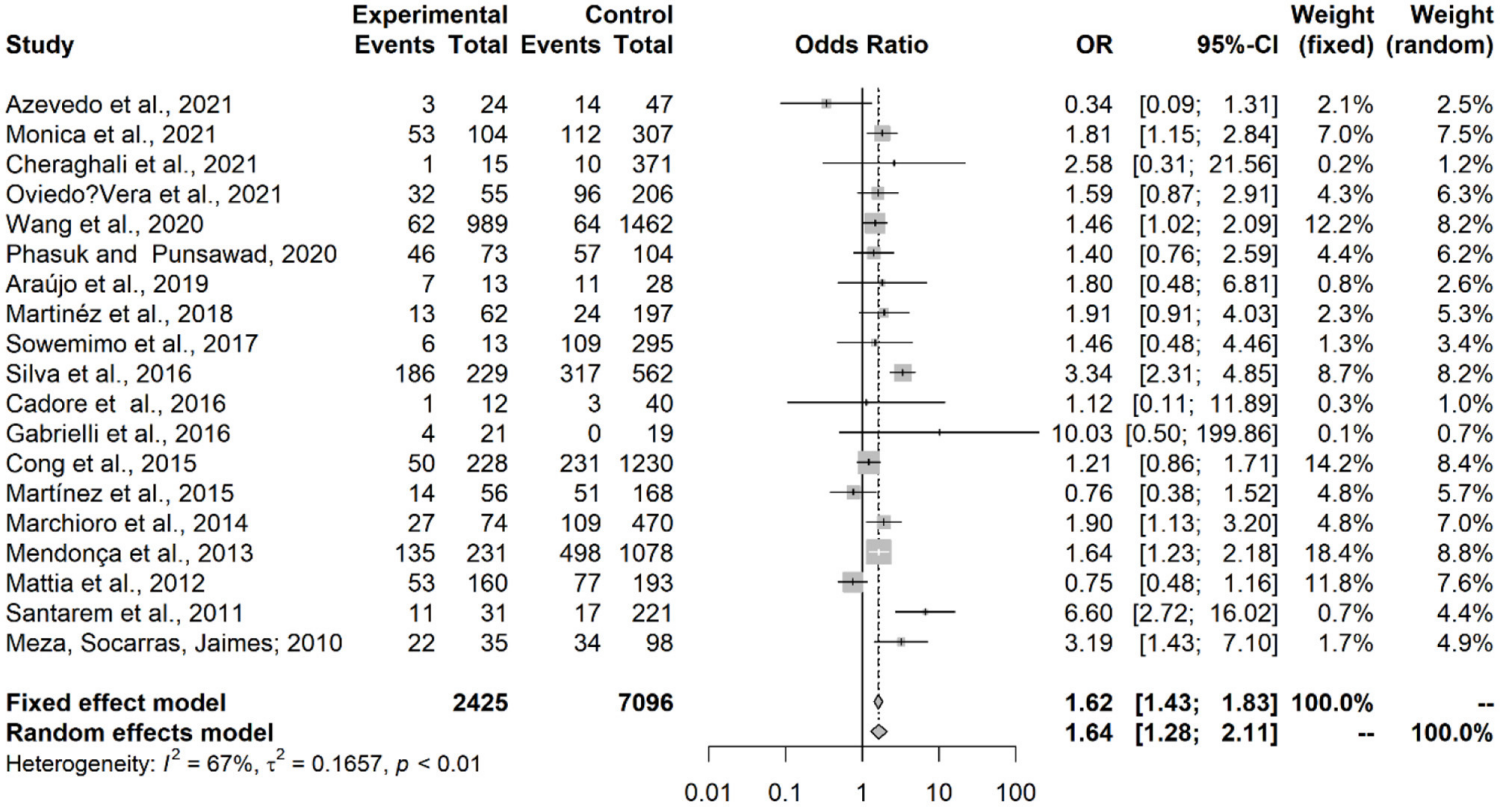
Forest plot for assessment of *odds-ratio* of influence of contact with cats in the frequency of anti-*Toxocara* antibodies in under-18 participants, according to selected articles used in the meta-analysis from 2009 to 2021.

**Figure 4:**
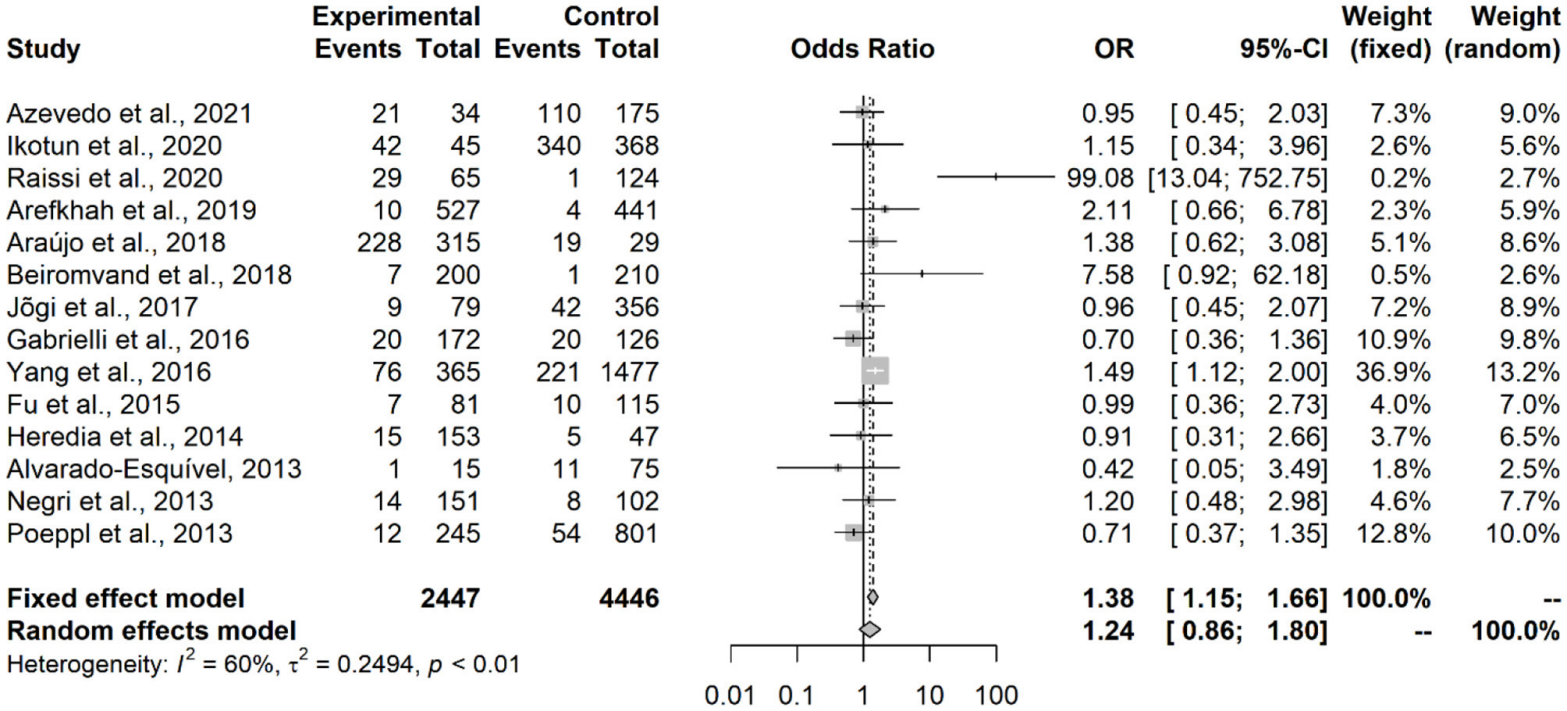
Forest plot for assessment of *odds-ratio* of influence of contact with dogs in the frequency of anti-*Toxocara* antibodies in adult participants, according to selected articles used in the meta-analysis from 2009 to 2021.

**Figure 5:**
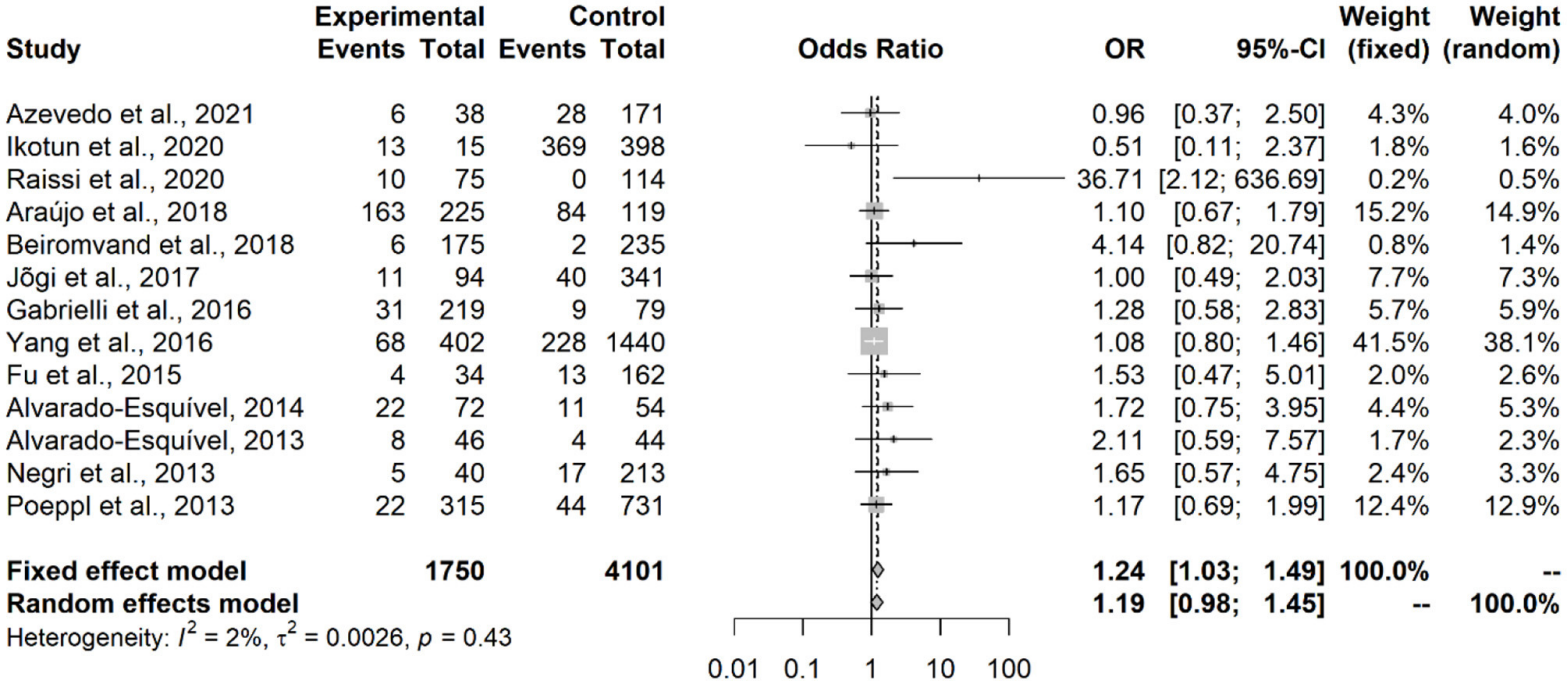
Forest plot for assessment of *odds-ratio* of influence of contact with cats in the frequency of anti-*Toxocara* antibodies in adult participants, according to selected articles used in the meta-analysis from 2009 to 2021.

Results showed that contact with dogs (OR = 1.53; IC = 1.27–1.86; *p* < 0.0001) or with cats (OR = 1.64; IC = 1.28–2.11; *p* = 0.0001) represented an associated risk factor to seropositivity in under-18 participants (Figures 2, 3). On the other hand, no statistical difference of dogs (OR = 1.24; CI 95% = 0.86–1.80; *p* = 0.2494) and cats (OR = 1,20; CI 95% = 0.98–1.45; *p* = 0.0735) was observed in adult participants (Figures 4, 5).

**Figure 6:**
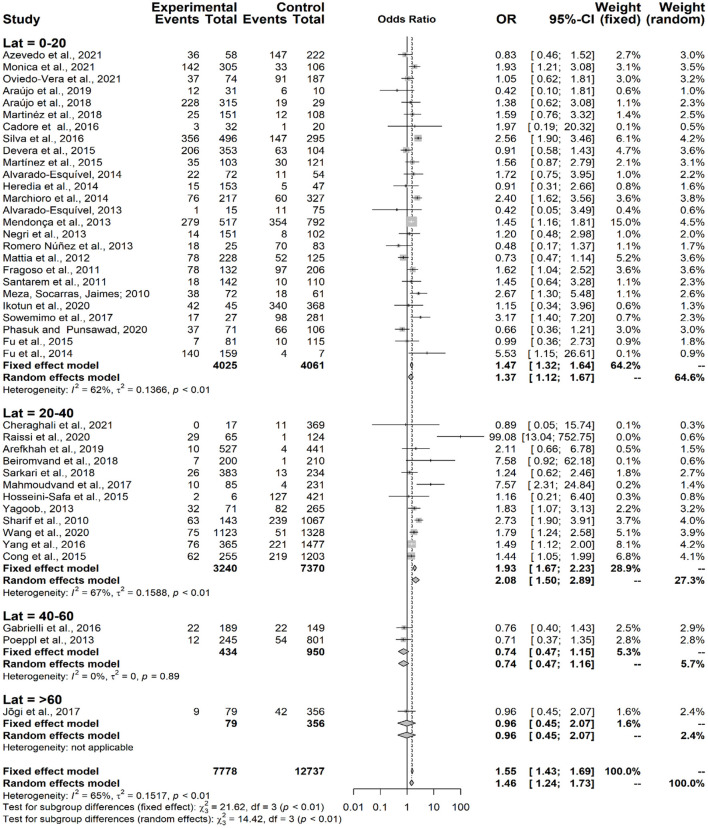
Forest plot for assessment of *odds-ratio* of influence of geographical latitude (Lat = degrees) in the frequency of anti-*Toxocara* antibodies in participants, according to selected articles used in the meta-analysis from 2009 to 2021.

The assessment of seropositivity for anti-*Toxocara* antibodies relative to the geographic coordinates showed that the higher the latitude, the lower the seroprevalence (χ^2^ = 14.42; *p* = 0.0024) (Figure 6).

**Figure 7:**
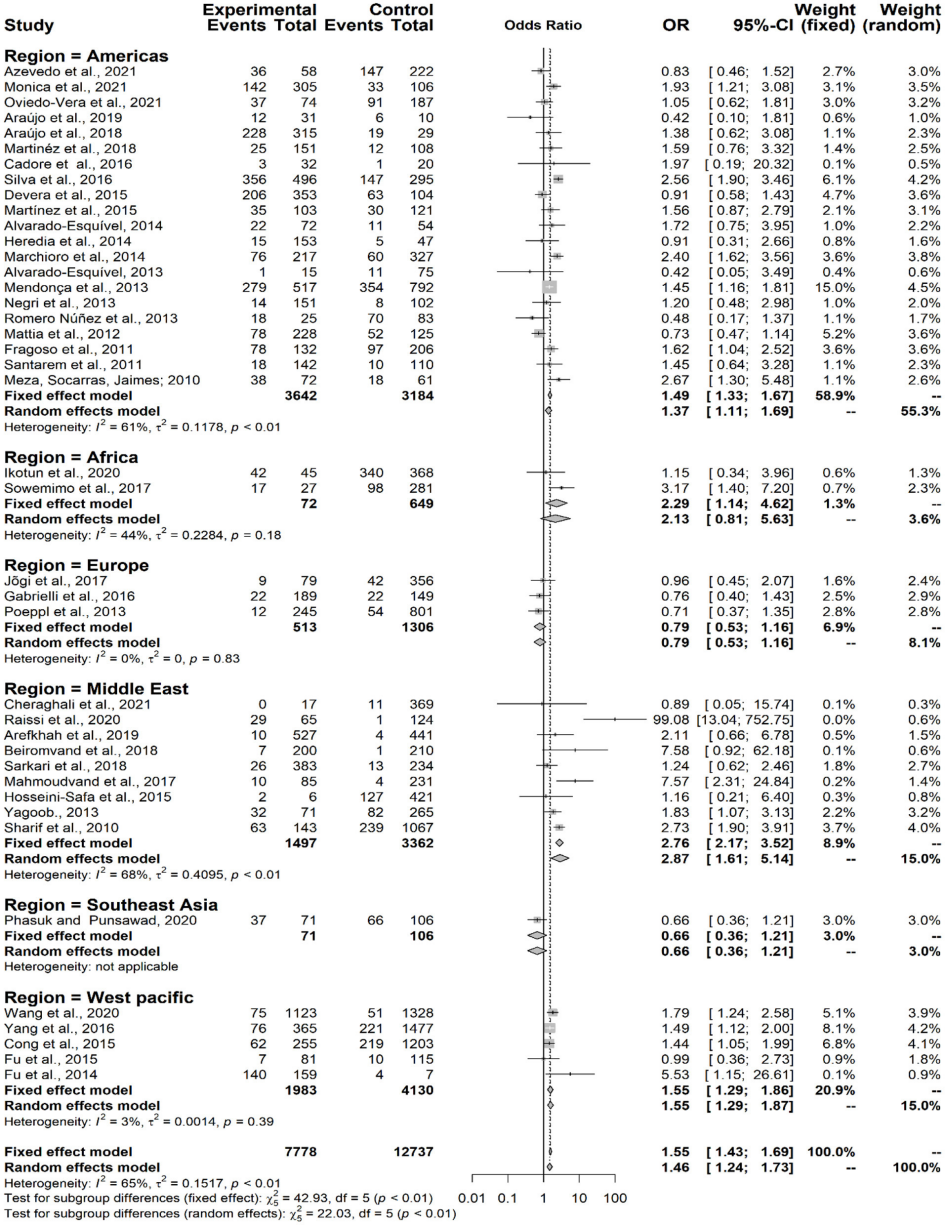
Forest plot for assessment of *odds-ratio* of influence of anti-*Toxocara* spp. antibodies in different world geographical regions (WHO- World Health Organization), according to selected articles used in the meta-analysis from 2009 to 2021.

**Figure 8:**
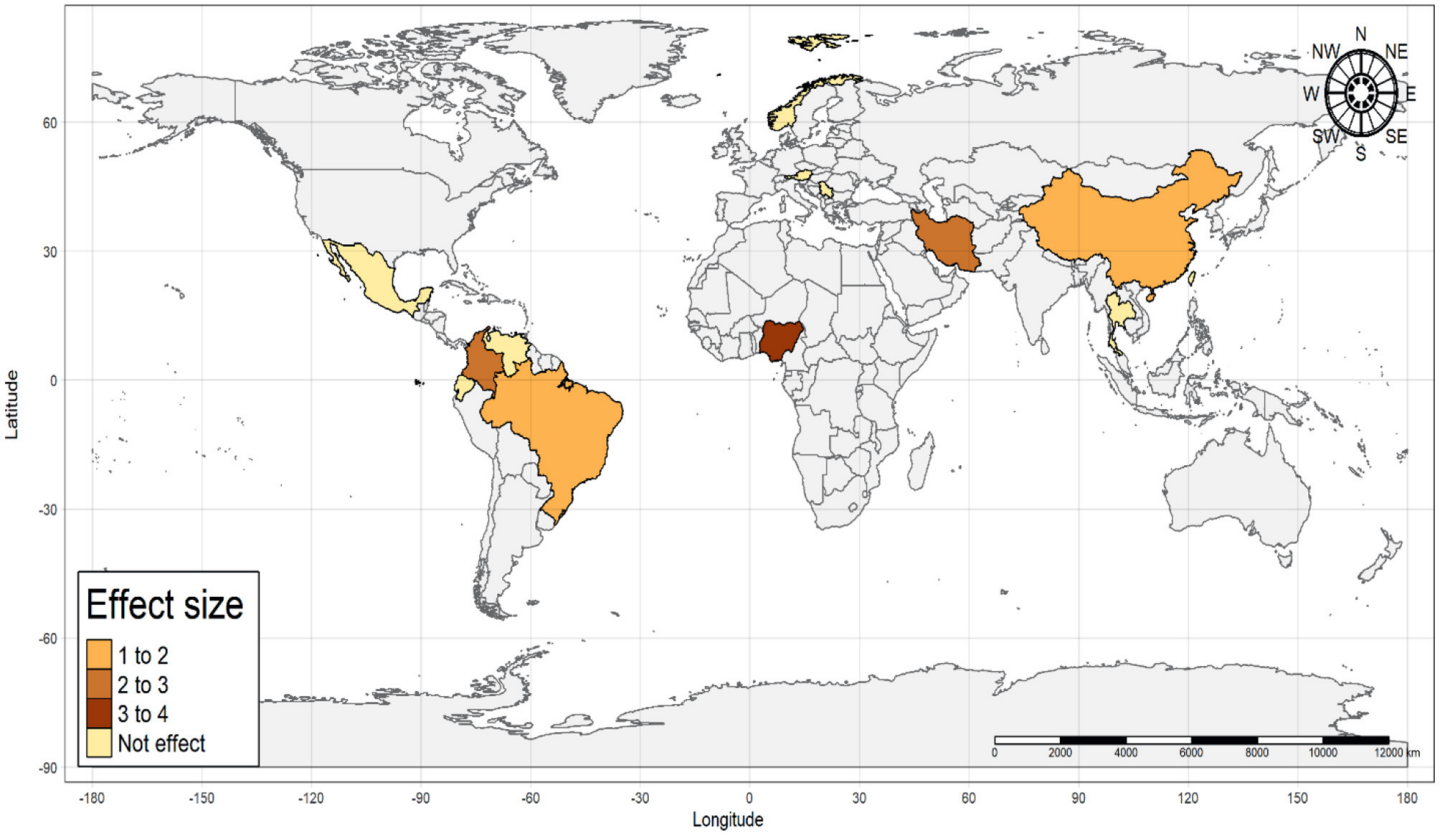
Illustrative map of effect size from studies on the frequency of anti-*Toxocara* spp. antibodies in under-18 and adults, according to selected articles used in the meta-analysis from 2009 to 2021.

**Figure 9:**
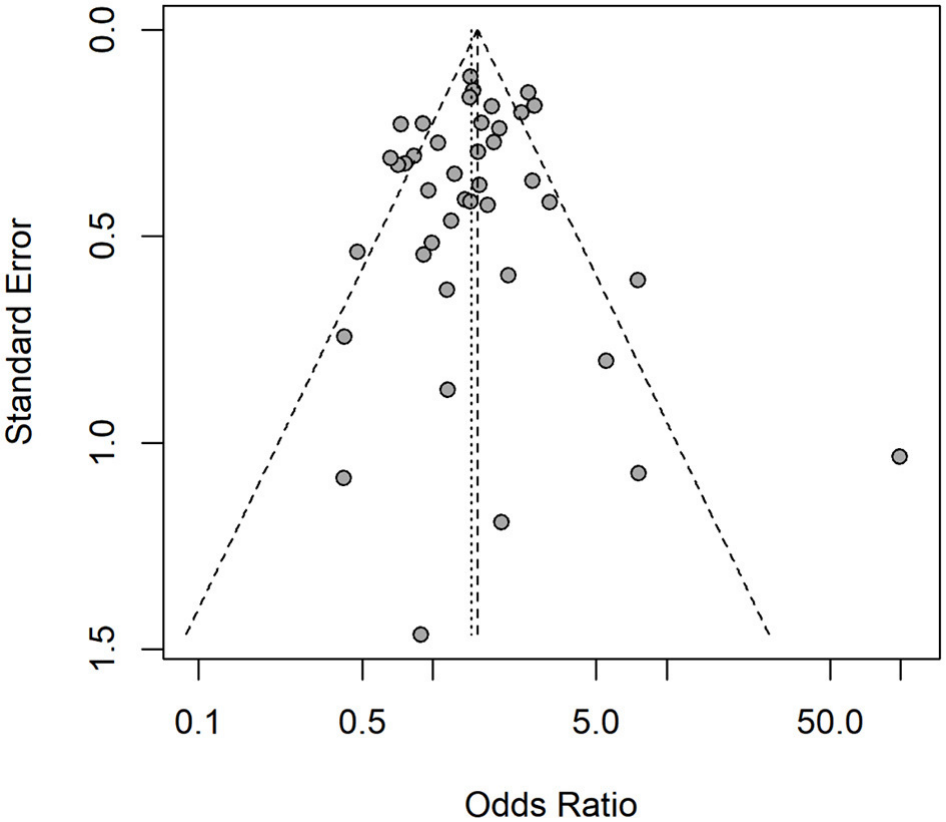
Funnel graphic utilized for assessment of publication bias, which has assessed the contact of dogs and cats and frequency of anti-*Toxocara* spp. antibodies in under-18 and adults, according to selected articles used in the meta-analysis from 2009 to 2021.

Considering the presence of anti*-Toxocara* spp. antibodies in populations from different geographical regions, according to the classification by the World Health Organization (WHO) (χ^2^ = 22.03; *p* < 0.0001), a statistically significant difference was observed in the Americas (OR = 1.37; CI 95% = 1.11–1.69), Middle East (OR = 2.87; CI 95% = 1.61–5.14) and Western Pacific (OR = 1.39; CI 95% = 1.03–1.88), but neither observed in Europe (OR = 0.79; CI 95% = 0.53–1.16) nor in Africa (OR = 2.13; CI 95% = 0.81–5.63), The single study from Southeast Asia has also no statistically significant difference (OR = 0.66; CI 95% = 0.36–1.21) concerning the presence of anti-*Toxocara* spp. antibodies (Figure 7). An illustrative map was created for a better pinpoint view of seroprevalence per country of study (Figure 8). The funnel graphic utilized for assessment of publication bias showed asymmetry among some studies, probably due to bigger size of effects and lower sample size (Figure 9).

Baujat plots showed that three studies (31–33) add substantially to the heterogeneity in the meta-analysis. However, these studies do not have a large impact on the overall results and were kept in the meta-analysis. One study was considered a potential source of publication bias, standing out as a point to the right in the funnel chart (31). However, it was found that this study has a substantially high odds ratio estimate compared to the others, more due to the low proportion of seropositive individuals in the control group than to a small study size. Thus, it was assumed that the effect size associated with this study does not imply a significant risk of publication bias.

## Discussion

The present meta-analysis study has found significant association between contact with dogs and cats and seropositivity for anti-*Toxocara* antibodies in under-18, but not for adult populations. Thus, contact with dogs and cats has been confirmed as an associated risk factor for *Toxocara* exposure in younger individuals. Based on the herein results, frequency of anti-*Toxocara* antibodies in under-18 with contact with dogs and cats (16.2%) was statistically higher than in adults (7.8%). Previous studies have shown that most seropositive youngers for anti-*Toxocara* antibodies were children from 2 to 8 years old with clinical history of onychophagia, geophagia, and exposure to animals (34–37).

A recent meta-analysis study has shown that frequency of anti-*Toxocara* antibodies in pediatric population worldwide was approximately 30% (IC = 22–37%: *I*^2^ = 99.11%; *p* < 0.05), similarly to the present study of 31.8% prevalence in under-18 individuals (26). Despite another up-to-date survey has also found youth as likely risk factor for toxocariasis (OR = 1.89; IC = 1.72–12.8), authors have not assessed presence of dog and cat contact as associated risk factor for youngers (4).

Children may be more exposed to toxocariasis agents due to habits of putting dirty hands into mouth, geophagia, and onychophagia (1, 38–40). Besides, children have a higher direct soil contact in recreation places such as parks and squares, which may be contaminated with *Toxocara* spp. eggs shed by dogs and cats (7, 41–43) Not surprisingly, one fifth of recreational public areas worldwide were contaminated with *Toxocara* spp. eggs (8). Another mechanism for *Toxocara* spp. transmission may rely on physical egg transfer from soil to owner shoes and animal paws, making even dewormed dogs and cats as potential helminth carriers (44).

Reportedly presence of *Toxocara* spp. eggs in dog/cat hair has been supported the hypothesis of transmission by ingestion of contaminated hair (13, 45, 46). Despite the contact with well-taken care dogs may represent low risk of infection, such potential transmission cannot be ruled out (11, 47). Children with contact with dogs or cats may present a higher tendency of acquiring infection by *Toxocara* spp. (48), with a higher risk of accidental intake of embryonated eggs of *T. canis* or de *T. cati* from contaminated pet hair (49). Daily contact with dogs and geophagia were the two most significant and influential factors for failure of toxocariasis treatment with albendazole in children from two to 16 years old in Poland, with prolonged treatment for toxocariasis more likely in children with daily contact with dogs (50).

The present study has found no association between contact with dogs or cats and presence of anti-*Toxocara* spp. antibodies in adults, which has been previously indicated as an occupational disease (51, 52). Moreover, toxocariasis in adults has been associated to consumption of non-treated water, raw and unwashed vegetables, raw or uncooked meat of paratenic hosts such as chickens, pigs, and rodents (53–55).

Regarding to the geographical regions, association of owning dogs or cats and be seropositive was observed in the Americas, Middle East and Western Pacific, corroborating to previous studies indicating areas of South America (27.8%) and Western Pacific (22.8%) with high frequency of anti-*Toxocara* antibodies (4). Besides climate conditions, socio-demographic factors such as low human development index (HDI), lack of veterinary assistance, pet outdoor access and risk of parasitic infection, and precarious self-hygiene may contribute altogether for a higher environmental exposure (1, 56, 57).

Although the frequency of anti-*Toxocara* antibodies in Middle East was relatively low, a statistical association was found with contact with companion animals. Besides the high temperature with low pluviometry in such region may be responsible for low environmental contamination and reduced seroprevalence (4), contact with dogs and cats may be limited due to religious reasons and legal restrictions for pet ownership (4, 42, 58, 59). On the other hand, the high feral, owned, and stray cat populations observed in Middle East countries such as Iran may favor infection risk due to contact with dogs and cats (20).

As a single study from Nigeria represented the entire Africa region, extrapolation of results and interpretations are limited and should be carefully taken. Toxocariasis studies in Africa have been mostly restricted and underreported (57, 60). In addition, seroprevalence studies such as in northern Africa (61) have not met inclusion criteria for the present meta-analysis, including case reports, lack of dog or cat assessment as associated risk factors, lack of population stratification, toxocariasis with comorbidities and potential bias in methodology, data extraction and/or interpretation. Despite report limitations, Africa may provide optimum toxocariasis transmission environment, including climatic settings, poor infrastructure, low socioeconomic conditions, and lack of veterinarian care (1, 56, 62). Such scenario strongly suggests that future studies should be conducted on prevalence and associated risk factors in throughout African countries as guidance for mitigation and prophylactic measures, as already proposed (57).

In Europe, no statistical association was found herein between owning a dog or cat and be seropositive for anti-*Toxocara* antibodies, which confirms previous studies showing that high income and HDI may present the lowest prevalence rates, consequence of fully access to information and prevention of infectious diseases, and to both human and animal health care (4, 63).

In Southeast Asia, only one study was included herein (64). This serosurvey evaluated children (5 to 15 years old), seroprevalence was 58.2% (103/177) and only the lack of handwashing before a meal was a significant risk factor (adjusted odds ratio (AOR) = 2.20; 95% CI 1.11–4.34; *p* = 0.023). Despite the high seroprevalence, interpretations of the results should be carefully taken since further investigation in Southeast Asia may provide robust data for a metanalytic investigation.

The observed trend of decreased frequency in anti-*Toxocara* antibodies related to increase of geographical latitude can be explained mostly due to colder climate of higher latitudes such as in Europe, unfavorable and limiting the life cycle of *Toxocara* spp. (4, 8, 65). On the other hand, countries located in low latitudes mostly present favorable climatic and environmental conditions to survival of *Toxocara* spp. eggs, associated to more non-dewormed stray dogs and cats in public areas (6, 8, 66).

As limitations, evaluated studies were incomplete and lacked information on sex of tested individuals, not allowing adequate assessment or comparisons of human gender involved on serologies. Thus, no assessment was made between contact with dogs or cats and gender of participants. In addition, few studies were included in the meta-analysis from certain regions, a single study from Africa and none from Asia, impairing an ideal analysis. Despite authors of studies in other languages, besides those included herein, were contacted to provide further information, no response was received back.

Studies included in the present metanalysis mainly used ELISA test for anti-*Toxocara* spp. antibody detection, mostly by *Toxocara* excretory-secretory (TES) antigens. As such specific IgG detection frequently persist for years, tests have not allowed differentiation between active and persistent infection (67). In addition, false-positive results in serological assays may occur in coinfections with other helminths due to cross-reactivity, as all Ascarid parasites may share a high homology of antigenicity with *Toxocara* spp. (68). Reduction of cross-reactivity in the *Toxocara* ELISA test has been obtained by preincubation with extract of adult *Ascaris suum*, removing antibodies elicited by exposure to Ascaris (10, 69). In addition, cross-reaction has been also reported between *Toxocara* spp. *Toxocara* spp. and *Trichinella* spp. (70), *Angiostrongylus cantonensis* (71), *Echinococcus* spp. (72). Consequently, seroprevalence assessed in metanalytic studies should be carefully interpreted to avoid under or overestimation due to differences in sensitivity and specificity among different serological methods, particularly in populations living in areas of endemic polyparasitism (4).

In summary, the present study has shown a statistical influence of contact with dogs or cats and serological exposure to *Toxocara* spp. in under-18 individuals. Such robust finding on associated risk factor strongly indicates special attention on preventive measures for toxocariasis, particularly to youngers in contact with dogs or cats. In addition, other measures such as preventive anti-helminthic treatment for dogs and cats, adequate removal and disposal of pet feces from parks and other public areas, population management of stray dogs and cats, and preventive educational programs for toxocariasis, particularly to youngers (4, 43).

## Data Availability Statement

The original contributions presented in the study are included in the article/Supplementary Material, further inquiries can be directed to the corresponding author/s.

## Author Contributions

YM, RG, and VS: conception or design of the work and final approval of the version to be published. YM: data collection. YM, RG, RS, LK, AS, AB, and VS: data analysis, interpretation, and drafting the article. LK, AS, and AB: critical revision of the article. All authors contributed to the article and approved the submitted version.

## Funding

This research was funded through the Araucária Foundation of Paraná State (Protocol # SUS2020111000010) to AB.

## Conflict of Interest

The authors declare that the research was conducted in the absence of any commercial or financial relationships that could be construed as a potential conflict of interest.

## Publisher's Note

All claims expressed in this article are solely those of the authors and do not necessarily represent those of their affiliated organizations, or those of the publisher, the editors and the reviewers. Any product that may be evaluated in this article, or claim that may be made by its manufacturer, is not guaranteed or endorsed by the publisher.
